# The Social Construction of “Emerging Elders”: Implications for Age-Friendly Community Assessments

**DOI:** 10.1177/2333721418784844

**Published:** 2018-07-20

**Authors:** Noelle L. Fields, Gail Adorno, Brandi Felderhoff, Rupal Parekh, MSW, Vivian Miller, MSW, Karen Magruder, Kellie Rogers

**Affiliations:** 1 The University of Texas at Arlington, TX, USA; 2 Providence Hospice of Seattle, Tukwila, WA, USA; 3 Texas Woman’s University, Denton, TX, USA; 4 University of Connecticut, Hartford, CT, USA; 5 The Senior Source, Dallas, TX, USA

**Keywords:** emerging elders, age-friendly, aging well, assessment

## Abstract

The term “emerging elders” has surfaced in age-friendly community assessment tools to denote a subset of older adults; however, limited guidance is provided on its application to aging populations. The goal of this study was to develop a data-driven conceptualization of “emerging elders” as part of an age-friendly community assessment. Adults, aged 55 years and above, were asked about their subjective meaning of “emerging elder” within the context of a larger study of aging well in a large U.S. metropolitan city. Using inductive and deductive methods, the researchers analyzed qualitative data (*N* = 38) collected from individual interviews with homebound older adults (*n* = 15) and participants of three focus groups (*n* = 23). Four themes suggest that emerging elderhood is related to chronological age, functional ability, transitions, and self-identity. Findings suggest that the term emerging elderhood may foster negative images of older adults consistent with Western cultural discourse, despite the positive connotations associated with “emerging elder” in indigenous and spiritual communities. Findings underscore the need to further refine age-friendly community assessments that take into account the social constructions ascribed to older adults and need for strategies to engage emerging elders in future research of age-friendly communities.

## Introduction

By 2030, more than 20% of residents in the United States are projected to be aged 65 years and above, compared with 13% in 2010, and nearly 10% in 1970 (Ortman, Velkoff, & Hogan, 2014), calling attention to the increased needs of the growing aging population. The needs of informal caregivers are also expected to grow as family and/or friends, many of whom are working full time, provide the majority of care to older adults and provide critical support for individuals “aging in place” (Anderson, Dabelko-Schoeny, & Fields, 2018; Kossek, DeMarr, Backman, & Kollar, 1993). As part of a larger community-based participatory study to evaluate the “age-friendliness” of one metropolitan city in Texas, the authors explored the experiences of adults aged 55+ years to understand their current and anticipated needs for aging in place and to explore the concept of “emerging elderhood.” Guided by the 2009 Aging Texas Well community assessment toolkit from the Texas Department of Aging and Disability Services (DADS; currently known as Texas Health and Human Services), the researchers explored the experiences of study participants and their perceptions of the concept of emerging elder. The purpose of this study is to gain an understanding of the meanings of this concept newly applied in the United States—emerging elder—from the perspective of older adults. The researchers also aim to provide insight for practitioners, professionals, and researchers who are developing age-friendly initiatives in their own communities, and who work or will work in settings that serve emerging elders.

## Theoretical Perspective

Social constructionism emphasizes the importance of context and time and how social and cultural influences contribute to individual meanings of experience (Berger & Luckmann, 1966). Age identities are socially constructed through changing notions of what being an “elder” means and how it should be understood as an idea. A social constructionist approach asserts socially defined expectations of age-related behaviors with which emerging elders are expected to conform (Fealy, McNamara, Treacy, & Lyons, 2012). How aging is understood varies both within and between populations and plays an important role in the structuring of social power. Thus, a theoretically informed understanding of aging is a critical part of processes of social development and social change (Appleby, 2010, 2011).

Recent research suggests that language matters when it comes to older adults (Fick & Lundebjerg, 2017). Words such as the “elderly” and the “aged” may “connote discrimination and certain negative stereotypes that may undercut research-based recommendations for better serving our needs as we age” (Lundebjerg, Trucil, Hammond, & Applegate, 2017, p. 1). Language may also influence subjective aging and how an individual perceives his or her own aging process, which is influenced by personal and social experiences, cultural values, and societal structures (Diehl et al., 2014; Westerhof & Tulle, 2007). Subjective aging has been shown to have an influence on health, health behaviors, and longevity (Westerhof et al., 2014). Because the choice of words and language is fundamental to the meanings of aging and older adulthood, the theory of social constructionism guided the research design as the study aims to elicit the subjective meaning of emerging elderhood as it applies to older adults to highlight the personal meanings of the concept of emerging elders from the perspective of study participants.

## Literature Review

“Emerging elder” is a new concept surfacing across literature streams; for example, academic (Morris, Mueller, & Jones, 2010; Roberts, 2009), gray literature (Clark, 2008; Garber, Timko, Kerka, Wilkins, & Hildreth, 2006; Pevny, 2014; Schachter-Shalomi & Miller, 1997), and age-friendly initiatives in the United States and Australia (Adorno, Fields, Parekh, & Magruder, 2015; Cuyahoga County Planning Commission, 2004; Fawcett, 2014; Garber et al., 2006; DADS, 2009). Clark (2008) refers to emerging elders within the context of church congregations, where the title of elder refers to a role in church leadership. The most frequent reference to the concept of emerging elder is found within the literature related to age-friendly communities, aging well, and aging in place. Little description, however, of who or what constitutes an emerging elder is evident in these studies (Cuyahoga County Planning Commission, 2004; Garber et al., 2006; Texas DADS, 2009). Some research suggests that emerging elders are associated with a chronological concept of age, yet studies are not consistent on what age ranges encompass emerging elders (Cuyahoga County Planning Commission, 2004; Fawcett, 2014; Garber et al., 2006).

A broader search of the literature reveals that emerging elderhood may capture functional, rather than chronological, concepts of age. For example, Pevny (2014) suggests that emerging elders are individuals transitioning from middle to older adulthood who have not yet achieved the respect or wisdom often attributed to elders. Additional research suggests that being an emerging elder is a “gift” accepted once a person has reached self-actualization, having established healthy relationships, acceptance of mortality, and a defined legacy through full-life review (Schachter-Shalomi & Miller, 1997). Alaskan Natives, capitalize the term “Elder” within their native script. The term refers to an honored life mentor or “wisdom keeper.” Emerging elders are described as not only having wisdom, but also being sensitive, and living a life full of harmony and balance, with strong commitments to community, culture, learning, and sharing their experiences (Roberts, 2009). These descriptions of emerging elders speak to a unique sense of acceptance and openness toward individual experiences and their impact on society at large, rather than solely focusing on chronological age.

These varied definitions provide evidence for the lack of conceptual congruence regarding emerging elders. These gaps in the literature warrant further study and point toward a compelling need for research engaging older adults in exploring and describing the concept of emerging elders.

## Method

The researchers utilized an exploratory design and a qualitative approach to collect data from older adults aged 55+ years as a part of a larger “aging well” study. A community-based participatory research (CBPR) approach was used to promote an academic-community partnership that encouraged joint participation in the development of the research design, implementation, analysis, and dissemination of the results (Macaulay et al., 1999; Israel, Schulz, Parker, & Becker, 1998). This approach allowed the researchers to engage older adults through the research process, encouraging community and lay involvement in the study, which was approved by the university’s institutional review board. Researchers recruited the study participants through several avenues, including through community contacts at Meals On Wheels, local churches, and senior centers in a large, metropolitan city in Texas. The participation of an older resident who served as a community liaison (CL) and functioned as lay research personnel for the study was key to the success of recruitment. The local United Way agency helped identify CLs who were long-term residents of the city with strong community ties, knowledge of the city, and who had deep connections to their respective cultural communities. The CLs included one resident from the African American community, one resident from the Latinx community, and one resident from the Vietnamese community. The CLs provided insight into the planning of the project, recruitment, and data collection.

To explore participants’ perceptions of emerging elders, the researchers employed a purposive sampling strategy to identify residents in the community aged 55+ years that represented both homebound individuals with limited functional abilities, and individuals with greater mobility and independence across various geographical areas of the city (by zip code). The sample included participants across age categories to capture the heterogeneous nature of the older population and because the researchers aimed to gain the perspectives of study participants living at different stages of aging, including those who could retrospectively discuss their own experiences of emerging elderhood. The final sample included individual interviews with homebound older adults (*n* = 15) and participants of three focus groups (*n* = 23). See Table 1 for sample demographics. The protocol for the study was reviewed and approved by the University of Texas at Arlington Institutional Review Board before the research was conducted (2014-0275). All participants provided written informed consent as part of the study.

**Table 1.** Demographic Characteristics of Sample (*N* = 38).

Age range, *M* (*SD*)	58-85 years, 70.7 years (*SD* = 7.6)
Age categories (years)	
55-64	11 (29%)
65-74	14 (37%)
75+	13 (34%)
Gender	
Female	28 (74%)
Male	10 (26%)
Relationship status	
Married/partnered	15 (39%)
Divorced/separated	9 (24%)
Widowed	10 (26%)
Single	4 (11%)
Race/ethnicity	
White	27 (71%)
African American	9 (24%)
Latinx	2 (5%)
Number of years living in city	
Range	1-69 years
*M* (*SD*)	31.6 years (*SD* = 16.1)

### Data Collection

The research team conducted face-to-face focus groups and individual interviews with adults aged 55+ years using a semistructured interview and a focus group protocol. Semistructured interviews allowed members of the research team to vary the order of the protocol questions to allow for a more natural conversational flow (Gibson & Brown, 2009). The interview protocols were created based on feedback from the CLs, the literature related to age-friendly communities, and the Aging Well community assessment toolkit (Texas DADS, 2009). Each interview or focus group lasted approximately 60 to 90 min.

To explore the meaning/s of emerging elderhood, the research questions broadly asked: What does the phrase “emerging elder” mean to you? Probing questions prompted more specific details about the meaning of emerging elderhood within the context of aging well and growing older. Researchers also engaged in active interviewing that allowed both the participant and the interviewer to reflect, discuss, and examine the interview experience (Creswell, 2012).

### Data Analysis

Interviews were audio-recorded, transcribed verbatim, and imported into ATLAS.ti version 7.2 software. The research team individually read through all of the transcripts and then convened to establish a preliminary coding framework structured around themes from the World Health Organization’s age-friendly checklist (2007; for example, transportation, housing, social participation, civic participation, employment, community and health services). Next, the research team coded the data independently using a deductive approach and then the research team subsequently established subcodes inductively. For example, the research team used open coding for themes that did not fit within the established framework and also maintained a code list throughout initial coding to track the continuous development of codes (Saldana, 2013).

The data specifically regarding “emerging elders” were then coded via initial coding, closely analyzing and comparing them (Saldana, 2013). The research team identified a total of 45 items coded as being related to “emerging elders” through this process. Following initial coding, the use of pattern coding allowed for the development of major themes from this particular portion of the data (Saldana, 2013). The constant comparative method was also used to categorize codes and to promote the emergence of themes (Miles, Huberman, & Saldana, 2014). Members of the research team conducted member checks with study participants and the CL to obtain feedback on the preliminary themes, to establish trustworthiness of the data, and to increase the credibility of the findings (Lincoln & Guba, 1985; Saldana, 2013).

## Findings

### Chronological Age

Most of the study participants had not heard of “emerging elder” and were first introduced to the concept as a part of our research. For many participants, the concept suggested a particular chronological age range that encompassed individuals who are mid-life and young old. “An emerging elder, I would probably say those individuals who are like 55 going on 60 . . .” Although participants differed in what ages they thought a person should be to be considered an “emerging elder,” several participants indicated that this included persons aged 55 years to 65 years, saying “I would assume that would mean somebody maybe in their 50’s” and “Someone who’s just turned 65.” Several participants also felt that it represented a chronological age; however, they did not define an age range but rather expressed that the term would apply to a generation of individuals: “Emerging elder. Yeah it just seems like it means getting old” and it means “ . . .just getting older, I guess.”

### Functional Ability

Many homebound individuals and focus group participants indicated that they felt being an “emerging elder” represented a person’s functional ability. Specifically, participants suggested such a person felt or acted “young” or “younger” than their chronological age or health might suggest, or that they were still capable of living in an independent lifestyle.

. . . .because I don’t feel like I’m there yet. . . Because, you know, people are living to be 100. And so I don’t, just, even though I’m a senior citizen, I don’t feel like a senior citizen. . . Well I’m very active, you know. Even though I have a lot of things going on, you know, with arthritis and stuff like that, I don’t feel old.

Someone that’s still independent maybe. . . in denial, so they’re the ones that are older and don’t think they’re older.

Many participants indicated that they did not yet feel as though they were an “elder” or were “older.”

What we see when we go to the gym. . . .I think I’m one of the more active maybe. . . .some of them I know are older than I am, so not all seniors are old and decrepit and disabled. So what is it, the new 30 is now 40 or 50? I’m choosing to think I’m the new . . .not all aging people are old and disabled.

Another participant felt similarly about her previous view of herself, compared with how she now views her age. Their self-perception, regardless of age, is what may have defined them as an emerging elder.

Right, that hasn’t met the physical challenges yet, to see what their life is going to be. They are probably, well, like I was, expecting a carefree, happy elder time. That is what I was expecting. And I expect that’s what emerging elders, because in the media we see these images of older people, like if they are 70 or 80, and people just go nuts if they look good. . . Emerging elder would be someone that looked young and acted young. That’s what it is.

In contrast, for some participants this concept signified their lack of functional ability or an awareness that they were not able to do the things they would like to, or once were able to. Their “emerging” status meant that they are losing capabilities that may be key to “successful aging.” The changes that their bodies experienced enhanced their personal self-awareness regarding their age-related abilities.

I guess, that means when you’re aware that you’re up there becoming aware of elder status. And some of us always say, it surely isn’t fun being a senior. Who knew? This isn’t the golden age like we thought it was going to be. And some of us, I don’t think I have ever heard my other say that. But I know some of the, some of my friends say, my mother used to say, getting older isn’t fun. And I thought, uh-uh, that’s not a very good thing to look forward to. . . I wish that I was more able-bodied. And that’s just one of those things. I’m thankful for the health I do have.

Many participants, while they did not see themselves as older, acknowledged that they were not as able-bodied as others who may or may not be comparable with them in chronological age.

We’re talking about aging, and there’s all kinds of aging. Some obviously, are housebound, wheelchairs, whatever and those are disabled in some respect, not necessarily mentally. . . I walk past the basketball court. . . and those people are running flat out. . . it makes me tired to watch them.

Other respondents felt strongly that functional capabilities should play more of a role in defining an emerging elder, rather than chronological age.

. . .I don’t know how you word, there are people that are my age that are a hell of a lot older than I am. . . And people that are younger than I am, I know for a fact, that walk stooped and shuffle their feet, and they sit at home and do nothing.

#### Disease model of aging

Several participants suggested that emerging elderhood connoted a disease model of aging. For example, one participant stated, “Am I emerging into my 80s? Am I emerging to the other side of dirt?” Another participant shared, “I’m aging, screaming and kicking, because there’s so many things I want to do.” Finally, a participant expressed “I know I am, [getting older] you know. I don’t want to be old because I know what’s coming . . .death.”

### Transitions

Participants developed another theme, which was that emerging elders might be experiencing a period of transition from middle adulthood to older adulthood. “. . . . going toward the steps of becoming like a senior is what they call us. Is that what it is . . .the stage?” Several participants indicated that they did not want to transition nor did they want to accept that they were becoming “old.” One participant indicated that perhaps they could be emerging into an identity or phase of life where they are less valued than they once may have been.

So what you got to do, you got to merge into old age. And it’s, sometimes it’s a hard transition, you know. Because a lot of old people don’t want to be old. And they don’t want to be considered as old. They want to have the same respect they had when they were younger, you know. And some of them don’t get the respect, you know. I mean, I guess that’s about the best way I can, I’m going to emerge into it. . . . You know, it’s a changing over. It’s like a metamorphosis.

One respondent felt as if there were socially sanctioned and defined benchmarks associated with this transition period. This emergence was seen as a rite of passage, signified by observable changes for an individual.

No, I think emerging, it depends, again, one can emerge being an elder from 49 to 50 when you get your first AARP card. You can emerge from, emerging elder when you become 65 and get Medicare. You can emerge, you know in every aspect.

#### Health crises

Many respondents also expressed that they became emerging elders when they were struck by a sudden health crisis. They indicated that perhaps this was not a slow transition but an immediate, acute transition; they were not chronologically older, yet as soon as this health crisis or event occurred, they felt that they immediately transitioned to older adulthood. The crisis signified their “emergence” into being an older adult. “No, I was living here, and I had a full-time job. . . All of the sudden, everything just hit the fan.” One participant shared the story of how a particular health crisis forced both him and his spouse to make the transition.

It simply means that, getting older. That’s all. . . .See her accident occurred a year ago, so in the last year we are emerging very much. . . .We were just having fun until, bang. And actually, her, she has a brain disease, but the brain disease was a very slow, progressive thing, and so we weren’t facing that. It hadn’t started kicking in really strongly. It caused the fall. It caused the accident. But it was like a wake-up, certainly. So we emerged about a year ago.

### Self-Identity

The final theme that emerged from the individual interviews and focus groups characterized emerging elders as a time of self-identity that includes one’s awareness of aging, sense of self as feeling “old,” and a sense of empowerment. Several participants expressed that emerging elders are a group that is making or advocating for change. “The boomers. . . .People taking charge for themselves.” This theme also signified for participants who they may be pushing the societally accepted parameters for how they should engage or behave and what it means to age: “I mean, I guess it means older people, elderly people having new resources. . . ”

Notably, several focus group participants described themselves as “active” elders rather than “emerging” elders. However, one focus group participant indicated that perhaps not all emerging elders are as active and that maybe this is what moves them to be defined as “older” adults (vs. emerging elders). Several focus group participants also reported that individuals must be intrinsically motivated to push his or her own limits, to be considered an active or emerging elder.

I think a lot of it is what is inside the person. . . People have got to have something inside themselves to do. . . There’s a lot of people who didn’t get there, started until they would say 80 or 85. I read these books on the cover, and it will say, she wrote her first bestseller at 85, you know. And they go on from there. It’s an emerging elder kind of thing would be, you can do it yourself, with what is currently out there? Now you wanted to do something socially useful. You’re still looking for a job. . . We’re busy all the time with whatever we want. . .

Several focus group participants discussed changing the norms and pushing the societally imposed boundaries for aging adults.

Respondent: I think it’s so important that we get rid of the stigmas about aging, period, and about the word elder and about the word senior. But and some, the, you know, all the surveys, the Pew research or whoever shows that what older people like to be called the best, I mean, if you have to put a label on us as older adult.

Respondent: . . .Elder, I like that. In fact, I named my little business New Elder. . . But I want to take the stigma away from aging period, because we’re all going to do it, when we hope, if we’re lucky.

Respondent: And hope to do it gracefully.

## Discussion

Similar to existing literature, participants suggested that emerging elders may be a distinct chronological group, typically representative of persons aged 55 years to 65 years (Garber et al., 2006). However, our study also suggests that emerging elders are not strictly defined by age, but rather by when an individual feels that he or she has self-actualized (Schachter-Shalomi & Miller, 1997). Moreover, participants described emerging elderhood as the process of transition from middle age to later life (Pevny, 2014).

Study findings also suggest that the term “emerging elders” may foster negative images of older adults consistent with Western discourse, despite the positive connotations of emerging elder in indigenous and spiritual communities. Participants reported that emerging elderhood involves a process of becoming older in terms of chronological age, which may happen gradually through chronic illness or suddenly through a crisis in health. Nearly all (individuals, focus groups) associated this transition with negative experiences and outcomes. Finally, emerging elderhood was primarily consistent with participants’ personal meanings of aging well, while being grounded in cultural values as well as the medical model about dependency in later life.

Age-friendly community assessments might consider using alternative and affirmative language to reach older adults. Extant research found that the term “elderly” has been reported to be ageist and discriminatory against the population of older individuals (Avers, Brown, Chui, Wong, & Lusardi, 2011). In a study conducted of individuals above the age of 65 years, persons who were older rejected the terms aged and elderly (Walker, 1993). Our study findings suggest that using preferable, respectful, affirmative language may encourage older individuals’ participants in community assessments. Research also suggests that words such as (the aged), elder(s), (the elderly), and seniors may be stigmatizing and could stereotype older adults in a negative manner (Lundebjerg et al., 2017). Findings from our research suggests that the use of language that is more favorable toward older adults may support their active participation in designing and contributing to age-friendly assessments.

Moreover, future research is needed to improve age-friendly toolkits such as the one utilized by the DADS to capture the range of needs for differing groups of older adults as a part of age-friendly community assessments. The landscape of older adults is becoming increasingly diverse as the population increases. Racial and ethnic minorities constitute the fastest growing segment of older individuals in the United States today (Ortman et al., 2014). LGBTQA (lesbian, gay, bisexual, transgender, queer and/or questioning, asexual) aging adult community members are a largely invisible subset of older adults who face barriers and challenges to aging and are also a growing subset of older adults (Choi & Meyer, 2016; Movement Advancement Project, 2018). Finally, immigrant elders have an array of life circumstances and have unique characteristics, which must be understood as this foreign-born population continues to increase (Population Reference Bureau, 2013). Emerging elder may take on different connotations based on cultural norms across this diverse body of older adults, calling for an increased need in research to accommodate this growing population.

Finally, this study is not without limitations that should be considered when interpreting and applying the results. First, this study drew on a small, purposive sample from a specific geographic region. Therefore, the findings cannot be generalized. The rich data generated through the interviews, however, allowed for the identification of important themes. Second, our sample lacked diversity and included predominantly Caucasian females. Future studies should seek to continue inquiry about emerging elders using larger, more diverse samples that will promote transferability and increased variability in functional ability and health status of participants. Furthermore, future studies might also utilize a mixed methods design to explore the issues related to emerging elderhood to capture both quantitative and qualitative data.
